# Factors influencing somatic cell counts and bacterial contamination in unpasteurized milk obtained from water buffalo in Bangladesh

**DOI:** 10.1007/s11250-023-03644-x

**Published:** 2023-06-16

**Authors:** Shuvo Singha, Fabrizio Ceciliani, Md. Mizanur Rahman, Mohammad Abdul Mannan, Salma Chowdhury, Sanjib Chandra Nath, Ovirup Bhushan Paul, Ylva Persson, Sofia Boqvist

**Affiliations:** 1 Department of Veterinary Medicine and Animal Sciences, Università degli Studi di Milano, Via dell’Università 6, 26900 Lodi, Italy; 2 Department of Physiology, Faculty of Veterinary, Animal and Biomedical Sciences, Sylhet Agricultural University, Sylhet, 3100 Bangladesh; 3 Department of Medicine and Surgery, Faculty of Veterinary Medicine, Chattogram Veterinary and Animal Sciences University, Chattogram, 4225 Bangladesh; 4 Udder Health Bangladesh, Chattogram, 4225 Bangladesh; 5 Sustainable Enterprise Project, Sagarika Samaj Unnayan Sangstha, Subarnachar, Noakhali, 3812 Bangladesh; 6 Buffalo Research and Development Project, Bangladesh Livestock Research Institute, Savar, Dhaka 1341 Bangladesh; 7 Department of Animal Health and Antimicrobial Strategies, National Veterinary Institute, 75189 Uppsala, Sweden; 8 Department of Biomedical Sciences and Veterinary Public Health, The Swedish University of Agricultural Sciences, 75007 Uppsala, Sweden

**Keywords:** Milk hygiene, Unpasteurized raw milk, Milk value chain, Somatic cell count, Staphylococci, Enterobacteria

## Abstract

Little has been published on the factors influencing the safety and quality of milk derived from water buffalo in Bangladesh. This study aims to describe the milk hygiene parameters and milk chain characteristics of unpasteurized raw milk sold to consumers in order to improve milk hygiene. A quantitative study design evaluated somatic cell counts, total bacterial counts, and specific gram-negative (Enterobacteria) and gram-positive (staphylococci) pathogens in 377 aseptically collected milk samples. Samples were collected at multiple nodes along the buffalo milk value chain: 122 bulk tank milk samples were collected at the farm level, 109 milk samples at the middlemen level, and 111 milk samples at the milk collection centers. In addition, 35 samples were taken from various milk products at the retail level. It was found that progressively increasing somatic cell counts and bacterial counts, including potential pathogens, occurred along the milk chain. A seasonal increase in spring was found, varying based on the farming system (semi-intensive versus intensive). Other factors included water purity and cleanliness of containers, mixing buffalo and cow’s milk, and the location of the water buffalo milk producer (coastal or river basin). This study demonstrated how improving udder health and milk hygiene along the water buffalo milk value chain would increase the safety and quality of water buffalo milk in the study area.

**Supplementary Information:**

The online version contains supplementary material available at 10.1007/s11250-023-03644-x.

## Introduction

Milk provides an excellent nutrient source for many people worldwide, particularly in low- and middle-income countries (Adesogan and Dahl, 2020). Demand for milk products is projected to increase by 1 % in the next decade, forecasting an increase of 1.7 % in global milk production (OECD/FAO, 2019). Cow milk dominates global production (81 %), whereas water buffalo is the principal non-cow dairy production species, contributing 15 % of milk output (Minervino et al., 2020). About 97 % of the buffalo population resides in Asia, with water buffalo being the primary milk source in South Asia (Hegde, 2019).

In Bangladesh, small-holder farmers dominate the water buffalo farming sector by utilizing fallow land and feed resources and providing income-generation opportunities (Habib et al., 2017). Water buffalo are increasingly reared in a free-range system (locally known as “Bathan”), followed by household subsistence systems in the coastal areas, sugarcane belt, and marshland of Bangladesh (Hamid et al., 2016; Sultana, 2018).

Milk and dairy products can be contaminated directly through lactating buffalo mammary gland intramammary infection (IMI) by bacteria such as staphylococci and Enterobacteriaceae during episodes of subclinical or clinical mastitis (Singha et al., 2021; Singha et al., 2023). Previous studies have identified the presence of different IMI-associated pathogenic bacteria in buffalo milk and milk products, including non-aureus staphylococci (NAS), enterotoxigenic staphylococci, *Staphylococcus* (*S.*) *aureus*, *Escherichia coli*, and other coliform bacteria (Bauzad et al., 2019; Al-Rudha et al., 2021; Singha et al., 2021). Bacterial contamination can also occur during milking and milk handling, transportation to milk collection centers, and during the processing and selling of milk products (Sanaa et al., 1993; Islam et al., 2018).

Bulk milk somatic cell count (BMSCC) is a key indicator of udder health, reflecting the milk quality at the herd level. In addition to BMSCC, other bacterial indicators, including total bacteria count (TBC), total NAS (TNAS), total *S. aureus* count (TSA), and total *Enterobacteriaceae* count (TEC), are important when ranking the hygienic quality of milk (Anderson et al., 2011; Berhe et al., 2020). The acceptable limit for TBC in Bangladesh is < 2 × 10^4^ CFU per mL of milk (Bangladesh Standards and Testing Institute (BSTI), 1009:^1982^). It has been shown that, for dairy cows, microbial contaminations vary over time, depending on the handling and processing steps taken along the milk supply chain (Mpatswenumugabo et al., 2019). Several studies have assessed hygienic quality along Bangladesh’s dairy cow milk chain (Khaton et al., 2014; Islam et al., 2016; Islam et al., 2018). However, to the authors’ knowledge, limited information is available on the hygienic quality of milk and dairy products along the buffalo milk chain. Therefore, this study aimed to describe milk hygiene parameters and the water buffalo milk chain characteristics of unpasteurized raw water buffalo milk sold to consumers.

## Material and methods

### Description of the study site and population

Rajshahi, Jamalpur, Mymensingh, Moulvibazar, Bhola, Dhaka, and Noakhali districts are the country’s most significant contributors of water buffalo milk (Faruque, 2000; Uddin et al., 2016) and were included in this study. The number of lactating buffalo per farm ranged from 1 to 46, with an average of 7.8. On average, daily milk production per farm ranged from 1.5 to 150 liters.

Water buffalo in Bangladesh are reared and milked under five small-scale production systems, depending on topography, vegetation pattern, and the seasonal availability of feed resources. Free-range systems, such as the bathan (a free-range system with approximately 500 buffalo per farm) and semi-bathan systems depend on the pasture’s seasonal availability. In the bathan systems, water buffalo depend on grazing on fallow pasture on islands from spring to autumn. In the semi-bathan systems, buffalo from the islands are shifted to the mainland from late autumn to winter for 3 to 6 months because of a lack of available pasture on the islands. Up to 20 buffalo are tethered and stall-fed in the household system under available housing facilities. In semi-intensive systems, approximately 150 buffalo are kept on pasture in river basin areas during the daytime and at housing facilities at night. On intensive farms, up to 170 buffalo are reared for breeding in zero-grazing systems using housing facilities (Uddin et al., 2016; Rahman et al., 2019; Samad, 2020).

**Fig. 1:**
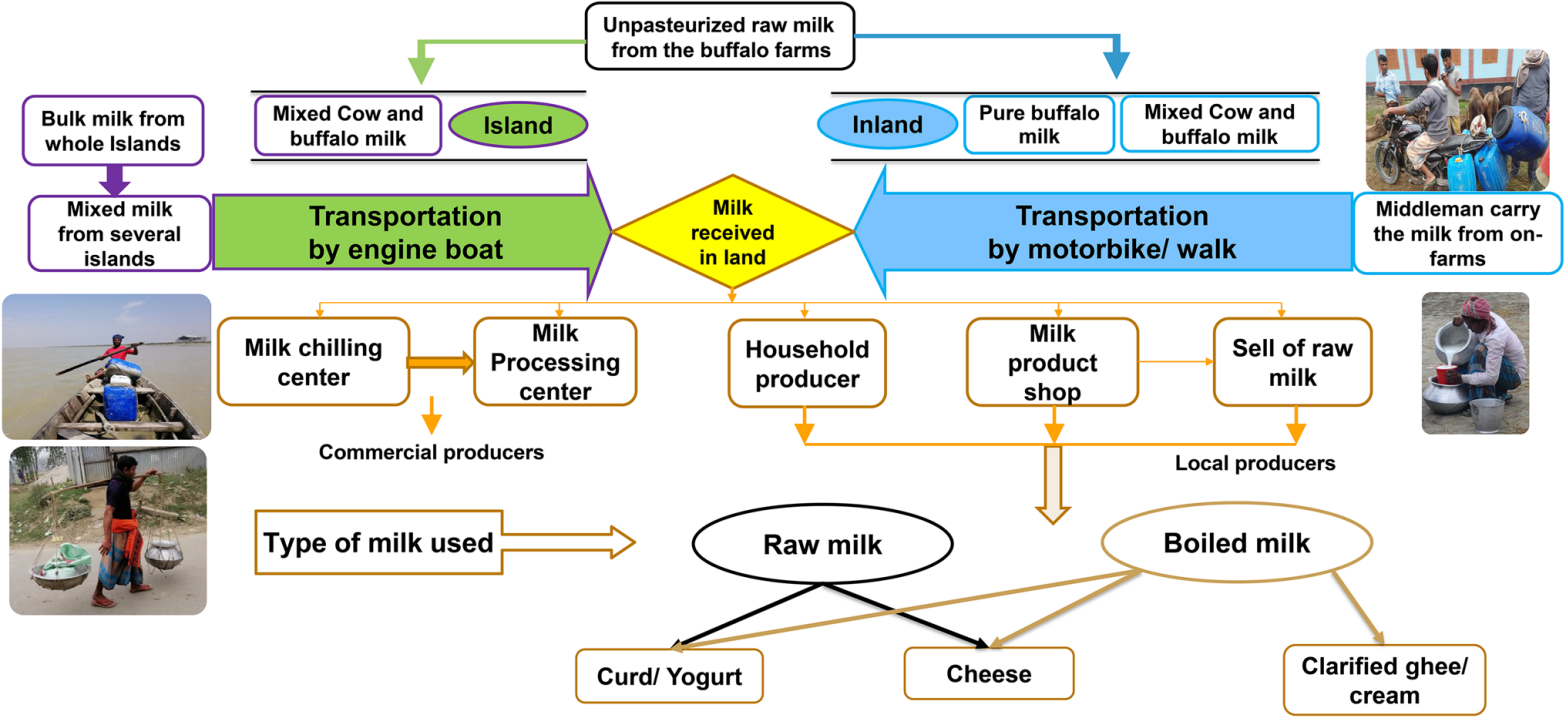
Schematic flow of the water buffalo milk supply chain in Bangladesh

Figure 1 shows the water buffalo milk supply chain in Bangladesh. The water buffalo milk chain in Bangladesh starts with small-scale milk producers, who perform the hand milking of the animals. Finally, milk is supplied to commercial milk processors or manufacturers through middlemen and milk collection centers (Hamid et al., 2016). Traditionally, the buffalo milk trade involves processing the milk into products like yogurt, cheese, and ghee, using raw or boiled milk without pasteurization. The most common buffalo milk product is yogurt, made from the natural fermentation of milk. Traditional hard cheese is made by acidifying milk using a lactic starter culture. Butter and ghee are the less popular buffalo milk products; butter is made by manually collecting the cream which settles at the top of boiled milk and then churning it. Ghee is then made by melting butter in a metal pan (Habib et al., 2021; Asif et al., 2022).

### Study design

**Fig. 2:**
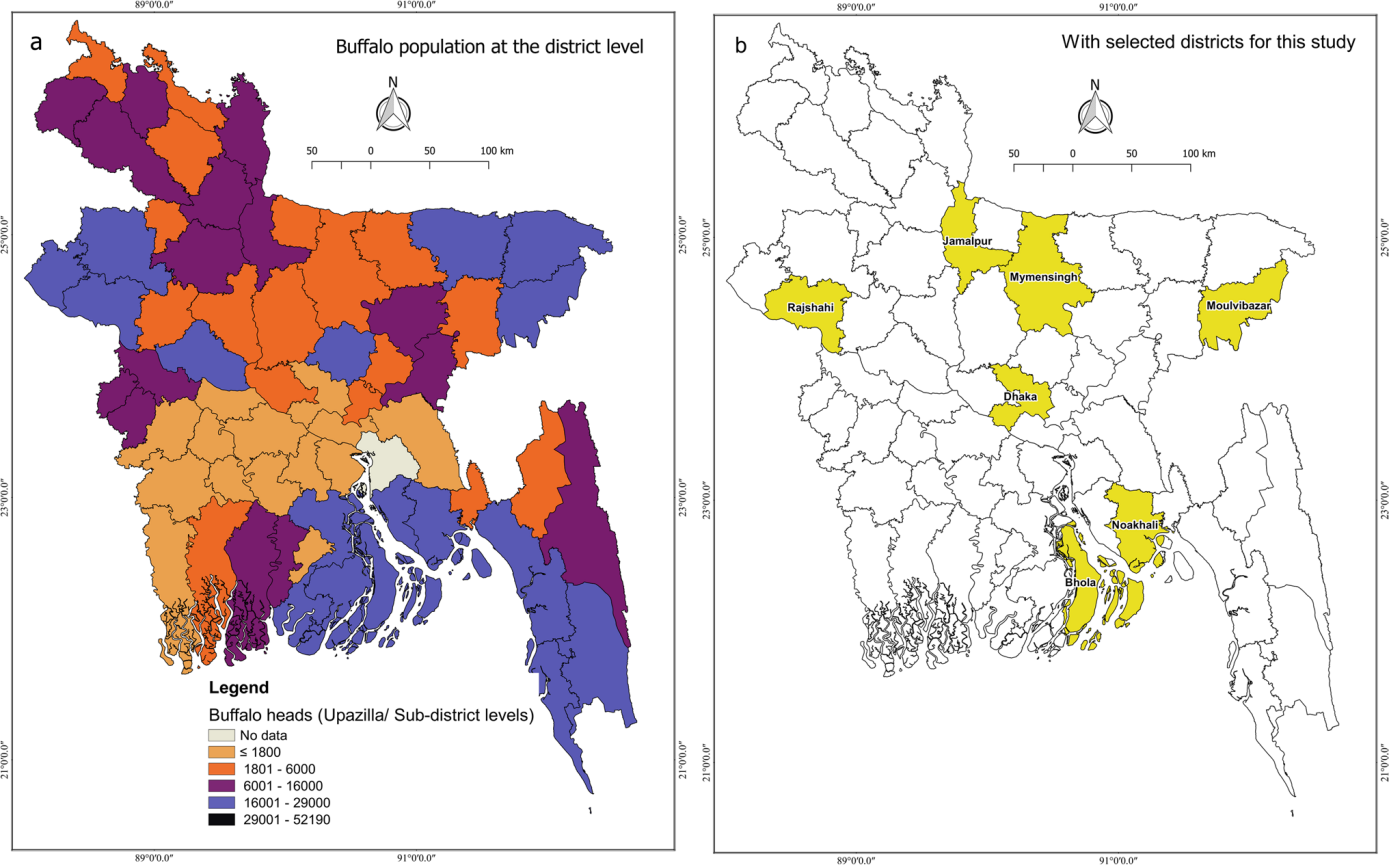
**a** District-level buffalo heads in Bangladesh are illustrated in the map of Bangladesh (Data source: LDDP (2019)). **b** The yellow area indicates the location of the study’s seven selected districts (Rajshahi, Jamalpur, Mymensingh, Moulvibazar, Bhola, Noakhali, and Dhaka)

Seven buffalo-concentrated districts mentioned in the section above (Fig. 2a and b) were enrolled in a cross-sectional study between February 2020 and April 2021. A list of the registered buffalo farmers was created with the help of the corresponding *upazila* (administrative unit of a sub-district in Bangladesh), veterinary hospital, and the non-governmental organization “Palli Karma-Sahayak Foundation.” The selected buffalo farmers were contacted with a request for information on the farm’s location and the middlemen and milk collection centers used. A total of 122 small-scale buffalo farms were recruited for this project. More detailed information about study design can be found in a study by Singha et al. (2023). No organized information, such as lists and contacts, was available for middlemen, milk collection centers, or milk product shops. Therefore, data collection from these nodes was carried out using the snowball technique (Etikan et al., 2016). One bulk milk sample was collected from each of the 122 selected farms, 109 milk samples were collected at the middleman level, and 111 milk samples at the milk collection centers (Table 1). Furthermore, 35 milk products, namely yogurt (*n* = 26), cheese (*n* = 7), and buttermilk (*n* = 2), were collected from milk product shops. Unique samples were collected from each included node, meaning the samples could not be traced back in the value chain. Epidemiological data was collected as described in the section *Collection of epidemiological data.*

**Table 1:** Distribution of milk and milk product samples (*N* = 377) collected at four different nodes of the buffalo milk chain in seven districts of Bangladesh. The bold entries in the bottom row indicates number of total observations for each column.

District	Farm	Middleman	Milk collection center	Milk products shop	Total number of samples
Noakhali	47	44	47	13	151
Bhola	18	18	18	10	64
Maulvibazar	16	16	15	5	52
Rajshahi	15	15	15	5	50
Jamalpur	17	11	11	2	41
Mymensingh	8	4	4	-	16
Dhaka	1	1	1	-	3
Total	**122**	**109**	**111**	**35**	**377**

### Collection of samples and handling procedures

After milking, the bulk milk was thoroughly mixed for 5 min, and 25–30 mL milk was collected aseptically in sterile 50 mL screw-capped falcon tubes from the top of the bulk. Two aliquots from the same farm’s bulk milk were collected, of which one was used to performing the BMSCC analysis, and the other was preserved aseptically for bacteriological culture. 10 mL bulk milk samples were collected aseptically from the middlemen nodes, and 10 mL of mixed milk samples were collected at the milk collection centers. The collected milk samples were not pasteurized. Approximately 30–35 g of milk products (yogurt, cheese, and buttermilk) were collected aseptically from each milk product shop in 50 mL sterile screw-cap bottles. All collected samples were transferred to an ice box immediately. Upon collecting all samples each day, the samples were frozen and stored at −20°C, and bacteriological quantification was performed 24 h after the samples were stored.

### Bulk milk somatic cell count of farm bulk milk

BMSCC was measured in the thoroughly mixed morning bulk milk using a DeLaval somatic cell counter (DeLaval Group, Stockholm, Sweden) (Adkins et al., 2017). Analyses were performed following the manufacturer’s instructions immediately after the collection of the bulk milk samples. A BMSCC level of 400 × 10^3^ cells per mL of milk, suggested by Costa et al. (2020), was used as the cut-off to compare the levels in the present study.

### Quantification of bacteria

Total bacteria, staphylococci, and Enterobacteriaceae counts were performed on a Plate Count Agar, Baird Parker Agar with egg yolk tellurite, and Violet Red Bile Glucose Agar, respectively. All agar media used were manufactured by Oxoid, Basingstoke, UK. To perform bacteria enumerations, 1 mL of the milk samples was mixed with 9 mL of diluent (sterile 0.9 % NaCl). Samples were serially diluted 10-fold up to 10^-7^. To estimate the total number of aerobic bacteria in the samples, the pour plate technique was carried out following ISO:4833-1 (2013). The staphylococci count was determined using the surface plate technique, following Viçosa et al. (2010). To confirm *S. aureus* colonies, five colonies were randomly chosen and tested using a coagulase test. The determination of Enterobacteriaceae was performed using the pour plate technique following 5^th^ ed. NMKL-144 (Nordic Committee on Food Analysis) standards. A further oxidase test was conducted on five randomly selected colonies to differentiate Enterobacteriaceae from non-Enterobacteriaceae. Bacterial enumerations were done considering a countable dilution containing < 300 colonies.

### Collection of epidemiological data

A questionnaire, divided into four subsections, was developed to collect data on factors potentially associated with farm BMSCC and bacteria from the buffalo milk chain nodes. Section A captured data at the farm level and included 45 questions. The data included information on farmers’ education level, buffalo rearing system, geographical area of the farm, the total number of lactating buffaloes, number of dry buffaloes, average milk yield per day, milking hygiene (excellent: milkers use antiseptic and wash hand; good: milkers only wash hand; poor: milkers don’t wash hand), and udder hygiene (excellent: udder is clean and dried; good: udder is clean but not dry; poor: udder is not clean and is wet). Section A also collected data on milk containers, such as the type of milk container, how the containers were cleaned, and the cleanliness score of the milking containers. The cleanliness score was defined using three categories, based on visual observation by the interviewer (excellent: no greasiness or dirt was observed inside or outside the container; good: no greasiness or dirt was observed inside the container; and poor: greasiness or dirt was present both inside and outside the containers). Data on milk storage, transportation, and their ability to obtain a reasonable price when selling the milk was also collected in Section A.

Sections B and C contained 20 questions and collect information from the middlemen and milk collection centers. Data on milk transportation (such as any cooling materials inside the containers), milk composition (buffalo milk or a mixture of buffalo milk and cow milk), how the containers were cleaned, and the cleanliness score of the milk container was collected.

Section D included eight questions to gather information on milk products, the type of milk used, storage time, and the type of containers used. Qualitative assessments, such as the cleanliness score of the milk containers, were determined during sample collection following subjective visual observation by the interviewer. Information on the type of milk was obtained by cross-questioning the middlemen or personnel at the milk collection centers.

Participation from the farmers, middlemen, and milk product shop owners was voluntary. Each farmer provided written informed consent to participate in the study. The middleman and milk product shop owners also gave written or oral informed consent. The study was approved and performed in line with the guidelines of the Sylhet Agricultural University Research System (AUP/21/06) in Bangladesh. The questionnaire was pretested and revised based on the comments from the pretest. Each questionnaire was given a unique identification number matching the identification number assigned to the collected samples at each level of the milk chain. The questionnaire is attached in a supplementary file (S1).

### Statistical analyses

Data from the questionnaires and the bacteriological enumerations were entered into an MS Excel spreadsheet. The data was cleaned, and coding and integrity were checked before importing the dataset into JMP 16.0 for statistical analysis (SAS Institute Inc., North Carolina, USA). BMSCC and bacterial count (TBC, TNAS, TSA, and TEC) data was log10 transformed to achieve normal data distributions. Descriptive statistics were performed using a boxplot for BMSCC at the farm level, and the bacteria count (TBC, TNAS, TSA, and TEC) at each node of the buffalo milk chain (farm, middleman, milk collection center, and milk product). A summary (mean and range) was presented for the quantitative variables, such as the number of lactating animals, the average daily milk yield at the farm level, and frequency numbers, with percentages calculated for the generic data, such as problems faced during transportation and storage, and farmers getting the right milk price.

Univariable analysis, a t-test, or a one-way ANOVA was performed to identify the variables (*P* ≤ 0.20) to be included in the multivariable regression models to investigate potential associations with BMSCC or bacteria contamination. Three multivariable regression models (Model-1 for BMSCC, Model-2 for TBC, and Model-3 for TSA) were constructed at the farm level and one at the middleman level to identify variables associated with the TBC. The models were built following a maximum likelihood estimation procedure and using a manual stepwise forward selection of the variables. Confounding was assessed by removing one variable from the model at a time and evaluating whether the coefficients changed by 30% and whether the confounding was biologically meaningful. Interactions were assessed by constructing two-interaction product terms for the significant main effects, adding them to the model, and examining changes in the *P* values of the main effects. The final model included variables with a *P* ≤ 0.05. A variation inflation factor and Cook Weisberg test were performed to identify multi-collinearity and heteroskedasticity.

## Results

### Bulk milk somatic cell count at the farm level

The geometric mean of BMSCC at the farm level was 254 × 10^3^ cells per mL; the highest value was 1.213 × 10^3^ cells per mL, and the lowest was 36 × 10^3^ cells per mL. Among the farms, 30 % (*n* = 37) exceeded the BMSCC threshold of 400 × 10^3^ cells per mL.

### Bacteria contamination at various nodes of the milk chain

**Fig. 3:**
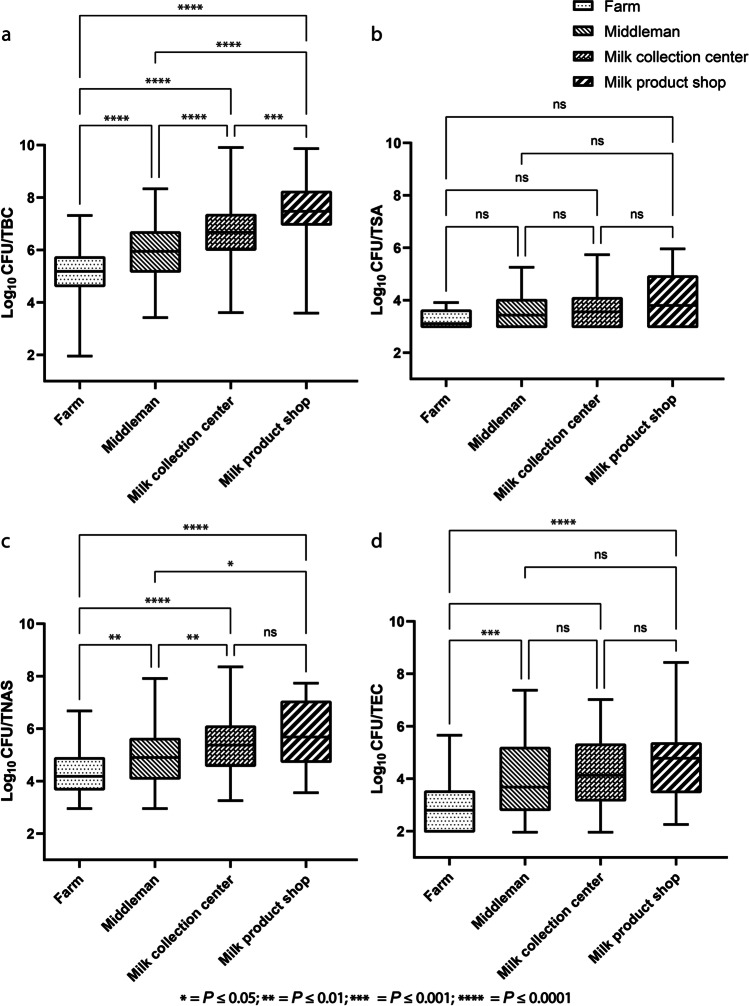
Assessment of bacterial contamination at four different nodes (farm bulk milk, middleman, milk collection centers, and milk products) of the water buffalo milk chain in Bangladesh (using box plots). **a** represents the total bacteria count; **b** represents the total *Staphylococcus aureus* count; **c** represents the TNAS count; **d** represents the total Enterobacteriaceae count. The horizontal lines show the comparison between the sample type, and the symbol for the significance level is indicated above the line. The level of significance corresponding to the symbol is displayed at the bottom of the figure

All the tested samples had a countable number of TBC, with 89–100 % being positive for TNAS, 48–80 % for TEC, and 13–18 % of the samples positive for TSA. A summary of bacterial counts (TBC, TSA, TNAS, and TEC) is presented in Table 2. A tendency towards an increase in TBC was observed along the milk chain (Fig. 3a, b, c, and d).

**Table 2:** Summary statistics of BMSCC and three different bacterial counts (per mL of milk) presented in log10 mean, range (minimum-maximum), and median for milk and milk product samples collected at four different levels on the buffalo milk chain in seven districts of Bangladesh

BMSCC/bacteria	Sample source (number of samples analyzed)	Number of positive samples (%)	Log10 mean (Min–Max) per mL of milk	Log10 Median per mL of milk
BMSCC ^**a**^				
	Farm (122)	-	5.4 (4.6–6.1)	5.4
TBC				
	Farm (122)	-	5.2 (2.0–7.3)	5.2
	Middleman (109)	-	6.0 (3.4–8.3)	6.0
	Milk collection center (108)	-	6.6 (3.6–9.9)	6.7
	Milk products (35)	-	7.5 (3.6–9.9)	7.5
TSA				
	Farm (122)	16 (13.1)	3.3 (3.0–3.9)	3.1
	Middleman (109)	15 (13.8)	3.6 (3.0–5.3)	3.4
	Milk collection center (111)	20 (18.0)	3.7 (3.0–5.7)	3.6
	Milk products (35)	5 (14.3)	3.8 (3.0–6.0)	3.8
TNAS				
	Farm (122)	111 (91.0)	4.4 (3.0–6.7)	4.2
	Middleman (109)	105 (96.3)	4.9 (3.0–7.9)	4.9
	Milk collection center (111)	111 (100.0)	5.4 (3.3–8.4)	5.4
	Milk products (35)	31 (88.6)	5.8 (3.6–7.7)	5.7
TEC				
	Farm (122)	58 (47.5)	2.9 (2.0–5.7)	2.8
	Middleman (109)	66 (60.6)	4.1 (2.0–7.4)	3.7
	Milk collection center (111)	85 (76.6)	4.2 (2.0–7.0)	4.1
	Milk products (35)	28 (80.0)	4.6 (2.3–8.4)	4.8

^**a**^*BMSCC*, bulk milk somatic cell count/ mL of milk; *TBC*, total bacterial count/ mL of milk; *TSA*, total *Staphylococcus aureus* count/ mL of milk; *TNAS*, total non-aureus staphylococci/ mL of milk; *TEC*, total *Enterobacteriaceae* count/ mL of milk

### Buffalo milk chain characteristics

Table 3 shows that a high number of the farmers (*n* = 53) had a primary education level (grade I-V) (46%), followed by farmers with no formal education (32 %) and farmers with a secondary (17 %) or graduate level (5 %). The farms used hand milking as their usual milking practice. About 79 % of the farmers did not use antiseptics and did not wash their hands before milking, 20 % washed their hands, and 2 % used antiseptics following hand washing. It was observed that, during milking, the udder was dirty and wet on 50 % of the farms; on 37 %, the udder was clean; and on 12 % of the farms, the udders were both clean and dry. None of the farms used pre- or post-milking disinfection. Of the farmers, 17 % sold raw milk from the farm to the local consumers, 70 % sold milk to middlemen, and 14 % sold their milk directly to the milk product shops. 34 % of the farmers responded that hand milking was problematic, as farmers were frequently injured during milking by the non-cooperative buffalo (Table 3). During transportation, 5 % of the middlemen mixed ice with the milk inside the milk containers, while the remaining did not provide any cooling of the milk. Most middlemen (64 %) cleaned the milk containers once daily, while 36 % cleaned them twice daily (Table 4). All the milk collection centers mixed buffalo milk with cow milk. The milk remained at the collection center for 0 to 6.5 h before further processing or sale. During milk storage, 96 % of the collection centers kept the milk at room temperature (25 to 30°C), while only 4 % stored it in freezers. About 63 % of the milk products were prepared in shops, and 37 % were prepared at the household level. The milk products were mainly processed using a mixture of buffalo and cow milk (80 %), followed by pure buffalo milk (20 %). Processed milk products were sold within 1 to 72 h (Table 5).

**Table 3:** Descriptive features of the farmers, hygienic practices, and herd population for 122 buffalo farms located in seven districts in Bangladesh

Variable name	Categories	Number (%)	Mean (Min-Max)	Median
Education level	No formal education	37 (32.0)	-	-
	Primary	53 (45.7)	-	-
	Secondary	20 (17.4)	-	-
	Graduation	6 (5.2)	-	-
Who milks the buffalo	Owner	72 (59.0)	-	-
	The owner, worker, and middleman	53 (40.9)	-	-
The score of milking hygiene	Excellent (use of antiseptic and hand wash before milking)	2 (0.02)	-	-
	Good (only hand wash before milking)	23 (19.7)	-	-
	Poor (no use of antiseptic or hand wash)	92 (78.6)	-	-
Score of udder hygiene	Excellent (udder is clean and dried)	14 (12.5)	-	-
	Good (udder is clean but not dry)	41 (36.6)	-	-
	Poor (udder is not clean and is wet)	57 (50.9)	-	-
Type of milk containers used	Aluminum	69 (58.0)	-	-
	Plastic	32 (26.9)	-	-
	Tin	13 (10.9)	-	-
	Others (aluminum, plastic, and glass)	5 (4.2)		
Water source for cleaning the milk containers	River water	25 (21.6)	-	-
	Tube-well water	57 (49.1)	-	-
	Pond water	34 (29.1)	-	-
Containers remain open during storing milk on the farm	No	113 (93.4)	-	-
	Yes	8 (6.6)	-	-
Point of milk sale	Farm	19 (16.5)	-	-
	Middlemen	80 (69.6)	-	-
	Milk product shops	16 (13.9)	-	-
Farmers age	-	-	36.7 (13–85)	35
Farm size (buffalo heads)	-	-	27.3 (3–170)	17
Numbers of lactating buffalo	-	-	7.8 (3–46)	5
Numbers of dry buffalo	-	-	6.6 (0–60)	3
Numbers of heifers	-	-	5.1 (0–95)	2
Numbers of calves	-	-	8.6 (0–51)	5
Average daily milk production per farm	-	-	13.6 (1.5–150)	8

**Table 4:** Descriptive features of milk handling, and hygienic practices by the middlemen (*n *= 109) on the buffalo milk chain in seven districts of Bangladesh

Variable name	Categories	Number (%)	Mean (Min–Max)	Median
Trading experience	Non-experienced (≤ 5 years)	2 (2.0)	-	-
	Experienced (> 5 years)	99 (98.0)	-	-
Cooling of milk	No	100 (95.2)	-	-
	Yes (Adding ice to the milk during transportation)	5 (4.8)	-	-
Materials used for covering milk container	Container kept open	63 (57.3)	-	-
	Cloth	1 (0.9)	-	-
	Plastic plate	32 (29.1)	-	-
	Aluminum plate	8 (7.3)	-	-
	Banana leaves	6 (5.5)	-	-
Nature of milk	Buffalo milk	62 (59.6)	-	-
	Cow-buffalo mixed milk	42 (40.4)	-	-
Cleaning frequency of milk containers	Once daily	65 (64.4)	-	-
	Twice daily	36 (35.6)	-	-
Water source used for cleaning the milk container	Pond water	49 (47.6)	-	-
	Tube well water	21 (20.4)	-	-
	Tube well water with detergents	33 (32.0)	-	-
Transport time (in hours) from middleman to milk collection center	-	-	1.5 (0–8.0)	1

**Table 5:** Descriptive features of milk handling, and practices associated with milk or milk product processing at the milk collection centers (*n* = 109) and milk product shops (*n* = 35) on the buffalo milk chain in Bangladesh

Milk chain nodes	Variable name	Categories	Number (%)	Mean (Min–Max)	Median
Milk collection center	Type of containers used	Aluminum	38 (36.9)	-	-
		Plastic	59 (57.3)	-	-
		Tin	6 (5.8)	-	-
	Stored milk in the freezing facility	Yes	4 (3.6)	-	-
		No	106 (96.4)	-	-
	Storage time (hours) before further processing	-	-	1.4 (0-6.5)	1
Milk product shops	Type of product	Yogurt	26 (74.3)	-	-
		Cheese	7 (20.0)	-	-
		Butter milk	2 (5.7)	-	-
	Product processing place	Shop	22 (62.9)	-	-
		Household	13 (37.1)	-	-
	Type of containers	Clay	12 (34.3)	-	-
		Plastic	16 (45.7)	-	-
		Glass	7 (20.0)	-	-
	Source of milk purchase	Own shop (own farm and through own contract middlemen)	10 (28.6)	-	-
		Both own farms, contract middlemen, and other farmers or middlemen	25 (71.4)	-	-
	Type of milk	Buffalo milk	7 (20.0)	-	-
		Cow-buffalo mixed milk	28 (80.0)	-	-
	Type of seller	Whole seller	9 (25.7)	-	-
		Retail seller	10 (28.6)	-	-
		Both	14 (40.0)	-	-
		Household seller	2 (5.7)	-	-

### Factors associated with bulk milk somatic cell count and total bacteria count at different nodes of the milk chain

Six independent categorical variables had a *P* ≤ 0.2 in the univariable analysis and were included in the multivariable analysis. Multivariable linear regression analysis at the farm level showed that in model 1, a higher BMSCC was observed in the spring season (*P* < 0.001) compared to the winter season and in the intensive buffalo rearing system (*P* = 0.006) compared to the semi-intensive system. In model 2, a higher TBC was associated with the winter season (*P* = 0.007) compared to the late autumn season, and milk containers were cleaned using pond water (*P* = 0.03) compared to tube-well water. In model 3, the TNAS count was significantly higher in the coastal or semi-coastal regions (*P* = 0.0003) compared to the farms in the river basin area (Table 6). Neither confounders nor interactions were observed in the models.

**Table 6:** Results from three multivariable linear regression models for the association between the log10-transformed bulk milk somatic cell count (BMSCC), total bacterial count (TBC), and total non-aureus staphylococci count (TNAS), with farm-level factors in Bangladesh expressed as co-efficient (β), 95% confidence interval (95% CI), and *P* values

Models	Variable name	Categories	β ± SE	95% C. I.	*P*
Model 1: Bulk milk somatic cell count ^**a**^					
		Intercept	4.86 ± 0.09	4.67 to 5.04	< 0.001
	Season	Winter	Reference		< 0.001
		Late autumn	0.14 ± 0.09	-0.04 to 0.31	
		Spring	0.51 ± 0.08	0.36 to 0.67	
	Rearing system	Semi-Intensive	Reference		0.002
		Household	0.18 ± 0.08	0.01 to 0.34	
		Free-range/bathan	0.21 ± 0.09	0.03 to 0.39	
		Semi-bathan	0.24 ± 0.07	0.10 to 0.37	
		Intensive	0.62 ± 0.17	0.27 to 0.97	
Model 2: Total bacteria count ^**b**^					
		Intercept	4.59 ± 0.18	4.23 to 4.95	< 0.001
	Season	Late autumn	Reference		0.018
		Spring	0.54 ± 0.20	0.14 to 0.93	
		Winter	0.63 ± 0.30	0.05 to 1.22	
	The water source used for cleaning the milk containers	Tube-well water	Reference		0.026
		River water	0.22 ± 0.24	-0.25 to 0.68	
		Pond water	0.50 ± 0.18	0.14 to 0.86	
Model 3: Total non-aureus staphylococci count ^**c**^					
		Intercept	3.98 ± 0.12	3.75 to 4.22	
	Geographical area of the farms	River basin	Reference		< 0.001
		Inland	0.38 ± 0.23	-0.08 to 0.84	
		Coastal or semi-costal area	0.87 ± 0.18	0.51 to 1.22	

^**a**^Based on records from 116 buffalo farms

^**b**^Based on records from 111 buffalo farms

^**c**^Based on records from 109 buffalo farms

A higher TBC in the milk samples from the middleman node was associated with cow and buffalo milk (*P* = 0.02) compared to pure buffalo milk. TBC levels were also significantly higher when middlemen used milk containers with poor cleanliness (*P* = 0.004) compared to those with excellent cleanliness (Table 7). No significant variables existed for the TSA, TNAS, and TEC models. No variables remained significant in the final multivariable models at the milk collection center and milk product shop level. All models fitted well and were free from any collinearity and heteroskedasticity.

**Table 7:** Multivariable linear regression analysis of the association between the log10-transformed total bacterial count (TBC), with middleman level factors in 102 middleman buffalo milk chain samples in Bangladesh expressed as co-efficient (β), 95% confidence interval (95% CI), and *P* values

Models	Variable name	Categories	β ± SE	95% C. I.	*P*
		Intercept	5.87 ± 0.12	5.64 to 6.11	
	Type of milk	Buffalo milk	Reference		0.02
		Cow-buffalo mixed milk	0.24 ± 0.10	0.04 - 0.44	
	Cleanliness of the containers	Excellent	Reference		0.01
		Good	0.006 ± 0.14	-0.27 - 0.28	
		Poor	0.47 ± 0.16	0.16 - 0.79	

## Discussion

This study showed moderate levels of BMSCC in milk from buffalo farms and high levels of TBC, TNAS, and TEC at various nodes in the milk value chain. The overall mean BMSCC at the farm level was 254 × 10^3^ cells per mL, which is higher than in a previous study (195 × 10^3^ cells per mL) in the Noakhali district in Bangladesh (Singha et al., 2021). A high BMSCC is a reliable parameter for indicating potential IMI in buffalo. A previous study reported that SCC in buffalo quarters substantially increases in the presence of an IMI, particularly when IMI is mainly caused by *Streptococcus* spp and *S. aureus* (Moroni et al., 2006). This indicates that IMI may be the most critical factor driving BMSCC, suggesting that improving buffalo udder health might reduce BMSCC.

This study found that the spring season and intensive buffalo rearing were associated with a high BMSCC level, confirming what has previously been reported in dairy cows and goats (Sargeant et al., 1998; Olde Riekerink et al., 2007; Koop et al., 2009). The buffalo included in this study were moved to the islands during spring, which might have added a level of stress related to the long transportation to the lactational stress as a response to the surge in milk yield, which may reflect a high BMSCC.

In the present study, the intensive buffalo rearing system had a large farm size (between 50 and 170 animals and generally had a high stock density), which may increase the chances of spreading IMI pathogens in the herd (Bari et al., 2022). Moreover, the animals lacked access to grazing and wallowing facilities, adding further stressful conditions and possibly compromising their immunity. The increased chances of IMI and stressful conditions may have led to a high BMSCC in the intensive farms included in the present study, given the correlation between high milk yield and increased BMSCC (Costa et al., 2020).

Buffalo farmers did not use BMSCC or any qualitative mastitis screening test, such as the California mastitis test. Therefore, milk quality in terms of udder health status from buffalo farms in this region of Bangladesh is mainly unknown. Currently, there is no regulatory enforcement for setting a threshold for BMSCC, and there are no dairy herd improvement programs like those found in the USA and Canada (Hand et al., 2012; Troendle et al., 2017). Therefore, the buffalo farmers in this study with high BMSCC are likely not motivated to upgrade their farm management based on BMSCC results.

The buffalo milk chain in Bangladesh is informal and has remained underregulated in terms of hygiene practices. This has been shown by the increase in bacterial contamination along the water buffalo milk value chain. The highest level of TBC was identified in the terminal milk chain nodes, e.g., milk products, compared to the lowest TBC levels observed in the farm bulk milk. The levels of TBC at the included nodes (5.2 log10 per mL at the farm level, 6.0 log10 at the middleman level, 6.6 log10 at the milk collection centers, and 7.5 log10 per mL in milk products) were much higher than the acceptable threshold of 4.3 log10 (2 × 10^4^) per mL set by the BSTI (1009:1982). This increased level of TBC in the terminal milk chain is consistent with a previous study from Bangladesh (Islam et al., 2018) and in studies from other low- and middle-income countries, such as Rwanda and Zimbabwe (Mhone et al., 2011; Ndahetuye et al., 2020), and is possibly related to mixing milk from different farms, bacterial growth due to long transport times in ambient temperatures, and unhygienic processing steps (Artursson et al., 2018; De Vries et al., 2020; Ndahetuye et al., 2020).

This study demonstrated that the winter season, as compared to late autumn, was associated with a higher level of TBC at the farm level. These findings can be partly explained by the fact that the transportation of the milk takes much longer in winter than in autumn due to water levels lowering, which makes boat transport difficult. A longer transport time might enable bacterial multiplication. On the other hand, in late autumn, the buffalo are kept closer to the location of milk collection centers on the mainland. This dramatically reduces the post-milking transportation time from the farm to the milk collection center, which may act as a protective factor in reducing bacteria contamination levels.

The use of pond water compared to deep tube-well water for cleaning the milk containers was also a risk factor, as has previously been reported (Aliyo et al., 2022). Ponds are generally located close to the manure reservoir, and using this water to clean the containers might cause bacterial cross-contamination (Lopes et al., 2021). At the middleman level, mixed cow and buffalo milk and a poor cleanliness score for the milk containers were significantly associated with high TBC, consistent with the findings of Aliyo et al. (2022). A mix of milk from different sources resulted in an overall high TBC level. Hence, from the findings of this study, cleaning the milk containers with clean tube-well water and avoiding mixing buffalo and cow milk could improve the hygienic quality of the milk.

NAS increased between the farm node (4.2 log10) and the milk products node (5.7 log10). Previous studies show that NAS species can colonize the teat canal of water buffalo during episodes of IMI (Singha et al., 2021), contaminating milk during milking. We identified higher levels of NAS associated with farms in coastal or semi-coastal areas, where buffalo farms are distantly located. Transportation depends on boats and walking, with the transport time to the milk collecting center reaching up to 6 h, promoting bacterial growth.

An increased level of Enterobacteriaceae was recorded in the milk products (4.6 log10) compared to the farm bulk milk samples (2.9 log10), as has previously been reported (Mhone et al., 2011; Knight-Jones et al., 2016; Islam et al., 2018), suggesting the fecal contamination of milk and confirming inadequate udder and milker hygiene on the farms. Regardless of the type of milk producers, there is a need to create motivation to follow the hygienic cleaning of milk containers to ensure safe milk for the consumers.

In conclusion, our findings suggest that better hygienic practices during milk handling could help reduce bacterial contamination and increase public safety when consuming buffalo milk and milk products in Bangladesh. To reduce bacterial contamination during transportation, the use of an insulated milk container with a cooling facility is advised. Milk from river areas was safer than milk from coastal areas, therefore further expansion of buffalo milk production could be focused on the river basins of Bangladesh. Also, introducing regular testing of BMSCC and implementing a penalty or premium system may help regulate the farms, thereby improving udder health and milk quality on the buffalo farms. It is also advised that microbiological evaluation of milk quality be enforced, and farmers could be encouraged by offering a premium price for higher milk quality.

In our study, the number of farms per district varied substantially, which resulted in a large variation in the number of farms per district in this study. However, most buffalo-concentrated districts were included in this study. Because of the similarity in the buffalo milk chain characteristics across the country, this study likely represents the overall udder health and milk quality of the buffalo milk value chain in Bangladesh.

## **Supplementary information**

ESM 1 **Supplementary file S1.** The complete questionnaire was used in the survey in this study. The questionnaire is divided into four sections that provide possible information relevant to bulk milk somatic cell count and bacteria contamination on the farm, middleman, milk collection center, and milk product retail level
